# Clinical Researches in Electro-Therapeutics

**Published:** 1873-06

**Authors:** A. D. Rockwell

**Affiliations:** New York, Fellow of the New York Academy of Medicine, Electoro-Therapeutist to the New York State Woman’s Hospital, etc.


ATLANTA

MEDICAL AND SURGICAL JOURNAL.

VOL. XI.—JUNE, 1873.—NO. 3.

---

### ORIGINAL COMMUNICATIONS.

---

### CLINICAL RESEARCHES IN ELECTRO-THERAPEUTICS.

BY A. D. ROCKWELL, M.D., OF NEW YORK,

*Fellow of the New York Academy of Medicine, Electoro-Therapeutist to the New York State Woman's Hospital, etc.*

A distinguished medical teacher remarked sometime since, before a large body of tyros in medicine, that electricity might be the coming remedy, but that in his experience it had utterly failed to be of the slightest service in the treatment of disease. Aided by a considerable personal experience, and by the patiently recorded cases of many conscientious and able collaborators in the field of electro-therapeutics and surgery, I can not, in weighing this expression, conclude otherwise than that the speaker was wofully deficient in a rightly guided experience in this department, or that he was blinded by an unreasoning and unreasonable prejudice. In whatever way it is regarded—whether in the believing or unbelieving mind—the remark must be stamped as unworthy a scientific man, unworthy because untrue. Scientific electro-therapeutics has had enough to contend against, both in the ignorant laudations of its real foes (that vast army of charlatans who for so many years have had no other thought in the culture of electricity than filling their own pockets,) as well as in the indifferent or contemptuous neglect to which it had been consigned by the great body of educated physicians.

It is not to be supposed that now there is any special fear for the future of electro-therapy. Like a tidal wave almost, the enthusiasm in regard to it has swept over two continents, and the only danger is that this marvelous reaction from a state of utter indifference will not be without attendant evils. In this way may injury be inflicted. The busy practitioner hearing on every hand vague rumors of the "marvelous cures" wrought by electricity resolves immediately to incorporate it in his own practice. Possibly, without comprehending even the distinctive physical differences between the various forms of the current, or the rationale, or the differential indications for their use, he procures with little thought a battery of some kind, and plunges at once in *medias res*.

He may at first, fortuitously, see results that wondrously elate him; but in the majority of instances those who attempt this department without some preliminary study will fail in the beginning, and the exceptional one who is successful at the start will receive in the end only a double disappointment? What is the result of this slip-shod method of procedure? He throws aside the battery, and even speaks of it in its relations to therapeutics in terms of contempt, forgetting that the man who regards his own failures and successes as a type of the failures and successes of all others, and deals with whatever contradicts his own individuality as if it contradicted human nature, is but a pedant and without true wisdom.

As in all other departments of our art, we are confronted in the practical study of medical and surgical electricity with failures that are sufficiently frequent, and oftentimes decidedly discouraging; and yet we may be assured that electricity has its place, and by no means an unimportant one, in therapeutics. To those who have given little if any attention to this subject, a plain statement of some of the main principles of electro-therapy may not come amiss.

Central galvanization and general faradization are the two methods to be used when it is desired to act generally on the nervous system, and to bring thoroughly under the influence of the current the various organs and tissues of the body.

Localized electrization, the third grand division of electro-therapeutics, is its own definition, and admits of an infinite variety of applications.

With the understanding that in a paper of the scope of the present one, it is impossible to give more than the most superficial glance at these methods, I propose to present a few cases as illustrative of their *modus operandi* and results:

*Cardiac Palpitation and Angina Pectoris—Decided Relief Under Central Galvanization.*

CASE I.—Mr. H. M., aged thirty-five years—referred to us by Dr. Leaming—had for twenty years, according to his own story, been a sufferer from cardiac palpitations, with some of the symptoms of angina pectoris.

The cardiac palpitations seemed to have a relation to the condition of the stomach, being associated with and apparently dependent on attacks of indigestion attended with regurgitation and pyrosis. It was one of those cases where it was difficult to determine precisely in what organ or nerve or nerve plexus the symptoms took their origin. The patient was not remarkably intelligent, but so far as could be gleaned from his history the attack involved both the heart and the stomach. It was certain that anything that excited indigestion brought on the attacks. Sometimes the paroxysms were ushered in very suddenly and with great severity.

Organic disease of the heart had been suspected, but Dr. Leaming, a skillful and practiced auscultator, decided that there was no such lesion. Medicine had accomplished but little for the patient, and we decided to use central galvanization.

The first application was mild and brief, but it caused much dizziness, and for the moment alarmed and worried the patient. In a few days he began to improve in his symptoms, and began also to bear the current better. For about two months the patient persevered in taking the treatment, with decided though not uniform improvement.

While the cure was not perfect, yet all his symptoms were so alleviated that life became in a measure enjoyable, instead of a heavy and constant burden.

*Unilateral Paralysis Agitans in a Man aged Sixty Years—Very Decided Alleviation of Symptoms uuder Central Galvanization.*

CASE II.—Mr. James A., aged sixty years. Suffering from unilateral palsy agitans of the left side. Was placed under our care by Dr. Andrews, of Utica, August 1st, 1870.

The first symptoms of this disease became manifest in October, 1869, and gradually increased in severity until January, 1870, since which time there had been no appreciable aggravation of his condition. The patient was a mechanic, occupied almost constantly in filing saws, and it was his firm impression that the peculiar influence transmitted to the cord by the steady scrape of the file was an important factor in the production of the disease. However this may be, it was certain that he was now unable to use his file a moment without causing most disagreeable feelings.

We essayed a few applications of general faradization, but as it did not seem to allay any of the disagreeable features of the disease, we resorted to central galvanization. After a few applications to the brain, sympathetic and spinal cord, the speech, which was decidedly affected, so that he stuttered and hesitated in every effort to talk, became perfectly normal, so that he was no longer annoyed by it. For many months he had been unable to sleep, unless lying on the back with the arms pressed to the side. In four weeks he was able to sleep with perfect comfort in any position.

The patient remained under observation some three months, and received in all about twenty-five applications. He improved in his general condition very decidedly; the arm and leg became much stronger, and the shaking movements decreased in severity at least fifty per cent. Further than this we were powerless to assist him.

In the above cases the method of procedure was substantially as follows: A sponge-covered electrode, about the size of a silver dollar and connected with the negative pole, was firmly applied to the epigastrium; and at the same time the positive pole, consisting of an electrode some two inches in diameter, was applied to the top of the head, or, as it is termed, the cranial centre. A current from three zinc carbon cells, of just sufficient strength to excite a slight metallic taste, was allowed to pass for half a minute, when the electrode was slowl[illegible] down the moistened hair of the back of the head until the sixt[illegible] cervical vertebra was reached. Here it remained for one minute, and was then carried slowly down the spine until it reached a point opposite the positive pole; the whole seance not exceeding five minutes.

Now, the operations in these cases are not minutely but only in a general way typical of the method of central galvanization. The length of the seances, the strength of the current, the position of the poles, etc., vary with the numberless varying circumstances that enter as factors into the history of the different conditions that we are called on to treat.

While it is impossible to enter into details, I would most earnestly caution those who may give this subject any attention, to be exceedingly careful in their first essays in their administration of central galvanization. Give much too little than a little too much. Let the first application be purely tentative and even completely negative in its results, rather than run the risk of doing harm. What is a small dose for one patient may be an overdose for another—sufficient not only to produce effects contrary to those desired, but capable also of causing decided injury.

Central galvanization is especially indicated in many cases of melancholia and hysteria, in neuralgias of central origin, in chorea, and in a wide variety of spasmodic conditions and forms of paralysis having their origin in the brain, spinal cord, or sympathetic.

*Hysterical Paraplegia After Slight Exertion—Complete Recovery Under General Faradization.*

Case III.—Miss J., aged twenty-five years. Was referred to us by Dr. C. M. Allen. The patient was of slight build, and her nervous system had from childhood been unduly excitable. Three years before, after an attack of typhoid fever, she observed an inability to walk more than a short distance without a sense of decided exhaustion in the legs. This symptom gradually increased in gravity until, in the course of a month or so, a minute's walk would be followed by violent trembling of the limbs, and such a feeling of loss of power that rendered further effort impossible.

[illegible]ing thus affected for a year, the patient visited Sara[illegible], where her condition very decidedly improved. The gain was, however, but temporary, for soon after leaving the Springs she relapsed, and for a year and a half every effort has failed to appreciably benefit her.

On the 4th of January, 1872, I began treatment by cautiously submitting the patient to general faradization. She was so susceptible to the influence of the current that the most gentle treatment was borne with difficulty. Gradually, from time to time, the power of the current was increased until she bore without discomfort very thorough treatment. After the first week slight improvement was manifested, which steadily continued until the 1st of April, a period of nearly three months, when she was able to walk any ordinary distance with perfect ease. The entire number of applications were forty. No relapse has occurred.

The modus operandi, rationale and effects of general faradization have been so frequently and so fully described that there is little, if anything, new to be now advanced. The case detailed above illustrates some of the effects of this method; in a general way its modus operandi may be given in a few words. The most thorough form of general faradization demands that the whole body shall be as thoroughly as possible brought under the influence of the current. The feet of the patient are placed on a copper plate, to which the negative pole is attached, while the positive, either the moistened hand or a sponge, is applied directly to all parts of the body from the head to the feet. A full and complete exposition of the subject of general faradization may be found in our recently published work.* The principle of the powerful constitutional tonic effects of electrization, when used in a general way, we enunciated more than six years ago. Time and experience have but served to more thoroughly convince us of its truth, and its value has been attested by thousands of well authenticated cases.

We very frequently meet with cases which, although they may be benefited up to a certain point by one or the other of the methods under consideration, do not completely recover. In such conditions we may often change the electric treatment, and

* Medical and Surgical Electricity. By Drs. Beard and Rockwell.

with most gratifying results. A case offers in which the indications unmistakably point to general faradization; under its influence the patient is decidedly benefited, and we look for a speedy recovery. Unexpectedly, however, improvement ceases or becomes barely perceptible. We now resort to central, and in some instances even to general galvanization, and the disease seems at once to take a new departure and disappears with surprising rapidity.

These conditions may be exactly reversed, being at first derived from galvanization, while faradization completes the cure. As a general rule, however, it is in cases where faradization fails, after benefiting to a certain point, that galvanization carries on to a successful issue the work begun.

If galvanization is really indicated in a given case, and improvement for a time follows its use and then ceases, it is only in rare instances that faradization can be of any possible service.

The two following are given as illustrative of this principle:

*A Case of Indigestion and Excessive Vomiting Associated mith Anæsthesia, Coldness and Weakness of Right Side—Approximate Recovery of Indigestion, etc., under Central Galvanization, and of the Anæsthesia, etc., under General Faradization.*

Case IV.—Miss C., aged twenty-three years. Came to us Oct. 15, 1870, with the following history: She suffered during childhood for several years from chorea of the right side, which at the age of twelve entirely disappeared. She then enjoyed fair health until the age of twenty-one years, when a condition of indigestion supervened that was fearful in its effects. Hardly anything could be retained upon her stomach, and during the winter she wasted almost to a shadow, and her life was despaired of. Under medical treatment and constant care her dyspeptic symptoms improved, and the vomiting was decidedly less. She increased in weight to her normal standard, and when we saw her she was able to retain the greater portion of the food ingested. Since the digestion began to improve, however, the left side of the body became markedly anæsthetic, cold, and greatly deficient in strength. The opposite side was slightly affected. General faradization, repeated six or eight times, almost completely relieved the symptoms of anæsthesia and decreased heat and strength, but had no special effect on the existing digestive disorders. Central galvanization, confined to the sympathetic upper portion of the cord and solar plexus, so far dissipated these symptoms that thereafter they annoyed her but little and only at long intervals.

*Spermatorrhœa Associated with Extreme Nervous Exhaustion of Three Years' Standing—Recovery under General Faradization and Central Galvanization.*

Case V.—G. H. W., a young man aged twenty-five years, came to us September 19th, 1871, complaining of a persistent spermatorrhœa, associated with great physical and mental depression. Three years before, he first observed a decided weakness of the eyesight, together with occasional nocturnal emissions of semen. It should be stated that these symptoms immediately followed a severe attack of inflammation of the bowels, with enlargements of the mesenteric glands. All his life he had indulged in masturbation to a considerable extent. His nervous system had been so completely upset that for three years he had been unable to study or to work, and as medication and the influences of travel and change had failed to benefit him, he began to despair of recovery, and became tenfold more despondent, and so reduced physically that he was unable to walk more than two or three short blocks without a sense of utter exhaustion and a soreness and "drawing down" in the abdomen that was absolutely painful. Seminal emissions occurred almost every night, and added immensely to his misery, both mental and physical. As an evidence of the excessively sensitive condition of the central nervous system, it may be stated that a faradic current of moderate tension applied to the sacrum and lumbar region produced by reflex action a decided tingling sensation in several remote parts, and especially on the crown of the head. The patient was submitted to general faradization, and under the influence of a dozen applications improved considerably in strength of mind and body. Subsequently many similar applications accomplished nothing more for him. Galvanization of the brain, sympathetic and spinal cord, was then resorted to. A new impetus seemed to be immediately imparted. The emission became less frequent, and finally occurred so seldom as to occasion little remark. The power of continuous thought returned, and at the end of another month he left us apparently recovered.

As already observed, localized electrization admits of a wide variety of applications, and includes, of course, the use of both currents. It may be thus sub-divided: 1st. Galvanization or faradization of the individual nerves, muscles and organs of the body; 2d. Galvanization of the brain; 3d. Galvanization of the sympathetic; 4th. Galvanization of the spinal cord; 5th. Cutaneous faradization; 6th. The electric moxa with either the galvanic or faradic current.

This system of electro-therapeutics and electro-diagnosis has been refined and developed from the following leading idea of Duchenne: He found that when dry electrodes were applied to the dry skins, sparks, with a dry, crackling sound, were produced, but no sensation and no muscular contraction. If one electrode was moistened, the other remaining dry, contractions, with the phenomenon of sensation, were excited under the moistened electrode; while if both electrodes were wet, muscular contraction and sensation were not only more decided but more deeply seated. *It was thus evident that electric currents could be localized over a fixed point under the skin, by strongly pressing well-moistened conductors upon the skin.*

*General Dropsy the Result of Valvular Heart Disease—Powerful Faradic Currents, localized, greatly increase the Secretory Action of the Kidneys, and Dissipate the Dropsical Effusion.*

Case VI.—December 13th, 1870, we were called to see, with Dr. Sam'l T. Hubbard, a lady aged about thirty-five years, who was suffering from general dropsy. The abdomen was enormously distended, and the lower limbs were double their normal size. The patient was a frail, delicate woman, and for years had suffered from valvular disease of the heart resulting from articular rheumatism.

The kidneys were almost entirely inactive, so that she voided not more than a teaspoonful of urine at a time, and the aggregate quantity secreted during twenty-four hours was but a trifle. All that we could hope to accomplish was to whip up the secretory process, and for this purpose a faradic current of great intensity was directed through both kidneys and the lower limbs. The current was to the patient hardly appreciable, notwithstanding the great strength of current used, and yet the flow of urine was so increased that during the next twenty-four hours a greater amount was voided than she was accustomed to pass when in her ordinary health. The applications were repeated fifteen times (the increased amount of urine secreted being kept up,) until the water had disappeared from the abdomen and legs. This was only one of several previous attacks, and her strength was so much reduced by continued suffering that she gradually sank and died. Electrization evidently prolonged life, and by relieving the pressure on the lungs, much alleviated the distress.

*Paresis of the Left Internal Rectus Muscle—Immediate Improvement under Localized Faradization.*

CASE VII.—Mr. Wm. B., with paresis of the left internal rectus muscle, was sent to us by by Dr. C. Agnew for electrical treatment.

The first symptoms of the difficulty dated some months back—just after his return from the west, where he had been subjected to unusual fatigue. A powerful current (faradic), localized as nearly as possible to the affected muscle, very markedly relieved the heaviness of the eyelid, and immediately improved the sight. For over a month the patient had been able to read only imperfectly and with difficulty, while an hour previous to the electrical treatment it was found on trial, at Dr. Agnew's office, that he was utterly unable to decipher newspaper print. Immediately after one application he read the fine print of the *Herald* with ease, and, in a day or so, a note from Dr. Agnew informed us that the vision of the patient had increased from one-tenth to one-half, and that the internal rectus had gained seventy—tested by prisms.

*Persistent Pain in and Contraction of the Flexors of the Fingers—The Result of Fracture of the Forearm—Immediate Relief follows Localized Faradization.*

CASE VIII.—Mrs. T., an old lady, was sent to us by Dr. W. H. VanBuren. While riding in a carriage during a European tour she was thrown out, and sustained a fracture of the forearm.

The bones readily united, but her fingers were left in a permanently contracted and painful condition, partly from the injury and partly from a rheumatic tendency. Localized faradization, together with galvanization, very greatly benefited her—dissipating in a great measure the pain and enabling her to open and close the fingers with comparative ease.

*Incipient Spinal Sclerosis Associated with Congestion—Apparently Permanent Arrest of the Rapidly Increasing Symptoms of Loss of Power, Incoördination of Movement, and Neuralgia, by Galvanization of the Spine.*

Case IX.—Mr. W. C., a gentleman aged thirty-six years, consulted us in July, 1870, for a condition which we diagnosed as spinal congestion accompanied by a more severe neuralgia of the legs than is usually associated with hyperæmia of the cord. The limbs were tender to the touch, and circumscribed patches, which were excessively sensitive, would suddenly appear and disappear in the lower extremities.

In addition, there was considerable decrease in the power of locomotion, attended by slight symptoms of incoördination of movement. These last symptoms were of such a character as led us to suspect incipient spinal sclerosis.

In a few months the patient had fallen away in flesh some twenty pounds. We employed in this case almost exclusively galvanization of the spinal cord, beginning with five, and as the treatment progressed, using as high as eighteen cells. The treatment was followed by complete relief of his severe neuralgic pains, increase in flesh, and decidedly more strength in and control over the legs. Before treatment the patient was rapidly growing worse. The result of the application was not to effect a perfect cure, since we believe that to be well nigh impossible, but the disease was at once arrested, and at this date, after the lapse of two years, the patient retains all the benefit derived from the dozen applications given.

